# A retrospective cohort study comparing controlled ovarian stimulation protocols for IVF/ICSI in women with endometriosis and diminished ovarian reserve

**DOI:** 10.3389/fendo.2026.1879193

**Published:** 2026-08-14

**Authors:** Fangfang Zhang, Yan Chen, Chen Lv, Fengjiao Ni, Lei Yan, Jialun Song

**Affiliations:** 1 State Key Laboratory of Reproductive Medicine and Offspring Health, Center for Reproductive Medicine, Institute of Women, Children and Reproductive Health, Shandong University, Jinan, Shandong, China; 2 National Research Center for Assisted Reproductive Technology and Reproductive Genetics, Shandong University, Jinan, Shandong, China; 3 Key Laboratory of Reproductive Endocrinology (Shandong University), Ministry of Education, Jinan, Shandong, China; 4 Shandong Technology Innovation Center for Reproductive Health, Jinan, Shandong, China; 5 Shandong Provincial Clinical Research Center for Reproductive Health, Jinan, Shandong, China; 6 Shandong Key Laboratory of Reproductive Research and Birth Defect Prevention, Jinan, Shandong, China; 7 Research Unit of Gametogenesis and Health of ART-Offspring, Chinese Academy of Medical Sciences (No.2021RU001), Jinan, Shandong, China; 8 Qufu Maternity and Infant Health Care Hospital;, Jining, Shandong, China

**Keywords:** assisted reproductive techniques, controlled ovulation stimulation protocols, diminished ovarian reserve, embryo transfer, endometriosis, infertility, pregnancy outcomes

## Abstract

**Background:**

Assisted reproductive treatment for women with endometriosis and diminished ovarian reserve (DOR) is challenging, and the optimal controlled ovarian stimulation (COS) protocol for this population remains uncertain. This study compared four commonly used COS regimens for IVF/ICSI in women with EM and DOR.

**Methods:**

In this retrospective cohort (2014-2023), 859 IVF/ICSI cycles in women aged 40 years or younger with EM and DOR were allocated to four COS protocols (ultra-long, long, short, antagonist). The primary outcomes were cumulative live birth rate (CLBR) and live birth rate (LBR) per started cycle; clinical pregnancy rate (CPR) was secondary. Between-protocol differences were assessed using multivariable logistic regression, adjusting for age, infertility duration, history of endometrioma cystectomy, basal FSH, LH, E2, AMH, antral follicle count, BMI, and COS protocol, with additional stabilized inverse-probability-weighting and 1:1:1:1 propensity score matching used to address confounding by indication.

**Results:**

The analysis included a total of 859 cycles, which were allocated into four treatment groups: the short protocol (n=295), the long protocol (n=145), the ultra-long protocol (n=202), and the antagonist protocol (n=217). Among these, the ultra-long protocol yielded the highest CLBR of 48.5%, LBR of 36.6%, and CPR of 41.1%. After PSM, the advantage difference of the ultra-long protocol is more significant. A subsequent multivariable logistic regression analysis confirmed that the ultra-long protocol was significantly more effective for achieving a live birth than the other protocols in the study: long protocol (OR: 0.577, P = 0.042), short protocol (OR: 0.608, P = 0.028), and antagonist protocol (OR: 0.486, P = 0.004). Similarly, the short (OR: 0.559, P = 0.010) and antagonist (OR: 0.484, P = 0.004) protocols were less effective for CPR.

**Conclusion:**

For infertile women aged 40 years or younger with endometriosis and DOR undergoing fresh embryo transfers, the ultra-long protocol appears to be more beneficial than the long, short, and antagonist protocols in terms of cumulative live birth, live birth, and clinical pregnancy rates.

## Introduction

Endometriosis (EM) is a multifaceted chronic inflammatory condition, and was reported to affect 5%-10% of individuals of reproductive age, and among individuals with infertility, the reported prevalence is 5%-50% (1). EM impacts female fertility through impaired oocyte quality, hormonal imbalances, and altered ovarian function (2, 3). In addition, the likelihood of detrimental pregnancy outcomes, including premature delivery, cesarean section, and placental abnormalities, may increase (4), with infertility being a common consequence, impacting around 30%-50% of women with EM (1, 5). Diminished ovarian reserve (DOR) refers to decreased oocyte quantity and/or quality, with a prevalence of 3.8% in women aged 20–24 and progressively increasing to reach 95.0% in women aged 45-49 (6). DOR is found in about 10% of the women seeking fertility treatments (6). Patients with EM frequently experience DOR due to the pathophysiological mechanisms of the disease, including the presence of ovarian endometriomas (7, 8). Compared with unilateral ovarian endometrioma cystectomy, bilateral cystectomy causes more substantial and enduring harm to the ovarian reserve (9).

Managing infertility in women with EM and DOR presents a complex challenge that necessitates careful consideration of the various treatment options, among which *in vitro* fertilization (IVF) is considered the most effective strategy. Although women with endometriosis may yield fewer oocytes and experience higher rates of cycle cancellation, these challenges do not significantly lower their ultimate pregnancy and live birth rates from IVF or ICSI procedures (10). However, compared to EM patients with normal ovarian reserves, individuals with DOR may have reduced oocytes, frozen embryos, and pregnancy rates. Therefore, an appropriate controlled ovarian stimulation (COS) protocol, particularly one suitable for fresh embryo transfer (ET), is crucial. Several studies examined the effectiveness of different COS protocols (8, 11, 12). However, women with DOR constitute one of the most challenging groups to treat effectively. A significant concern is that, despite intensive Gn stimulation, patients with DOR produce a low yield of accessible follicles (13). Despite the prevalence of EM and DOR, comparative studies directly evaluating COS protocols in this population keep limited.

Therefore, this study aimed to examine the reproductive outcomes across four COS protocols, namely the ultra-long, antagonist, long, and short protocols, in patients ≤ 40 years of age with infertility related to EM and DOR managed with assisted reproductive technology (ART).

## Methods

### Study design

From January 2014 to December 2023, this study reviewed the medical records of women who underwent IVF/ICSI treatment at the Reproductive Medicine Center of Shandong University. The study focused on patients with both EM and DOR. The institutional ethics committee approval number for this study is 2022-59. Due to the retrospective nature of this study, the ethics committee did not require patients to provide personal informed consent.

Inclusion Criteria: To be included, patients had to be ≤ 40 years of age and have a clinical or surgical diagnosis of EM (14, 15). Specifically, DOR was diagnosed if, over the span of two consecutive menstrual cycles, during the 2^nd^ or 3^rd^ day of menstruation, follicle-stimulating hormone (FSH) levels were ≥10 IU/L or vaginal ultrasonography showed an antral follicle count (AFC) of five or less, or serum anti-Müllerian hormone (AMH) levels were <1.1 ng/mL.

Exclusion Criteria: Patients were excluded from the study if they had uterine malformations, a history of recurrent miscarriage, were undergoing cycles that required preimplantation genetic testing for aneuploidy (PGT-A), or had polycystic ovary syndrome. Cycles with incomplete records were also excluded. In cases where a patient had undergone more than one ART cycle, only the first cycle that met the study’s criteria was included in the analysis.

### COS protocols for IVF/ICSI

This study assessed four commonly used COS protocols in ART. Oocyte retrieval was scheduled for 36–38 hours after the final hormonal trigger (16). The specific protocols used were as follows:

Ultra-long protocol: This involved administering a single 3.75 mg dose of a GnRH agonist between days two and three of the menstrual cycle. A follow-up visit was then scheduled to occur 28 to 35 days later.

Long protocol: This protocol began in the mid-luteal phase with the daily administration of 0.05 to 0.1 mg of a GnRH-agonist. A follow-up assessment was scheduled 14 days after treatment began.

Short protocol: On the second or third day of the menstrual cycle, treatment began with 0.05 to 0.1 mg/day of a GnRH-agonist. Ovarian stimulation then commenced within the following one to two days.

The antagonist protocol was initiated with Gn administration on days 2–4 of the menstrual cycle. A GnRH antagonist (cetrorelix acetate or ganirelix acetate) was introduced when any of the following criteria were met: serum estradiol (E2) levels exceeding 200–300 pg/mL, LH levels greater than 10 IU/L, or the presence of a dominant follicle measuring 11–12 mm in diameter. Final oocyte maturation was triggered when at least two follicles reached a diameter of ≥18 mm, using either a GnRH agonist trigger or a dual-trigger approach. Oocyte retrieval was subsequently performed 36–38 hours after trigger administration. The choice of trigger strategy was based on ovarian response characteristics. Patients with a high ovarian response (e.g., ≥15 follicles measuring ≥14 mm on trigger day or elevated serum estradiol levels) were considered for dual trigger strategy.

Within 2–3 days after retrieving the oocytes, two cleavage-stage embryos were implanted into the uterus with abdominal ultrasound guidance, or a single blastocyst was transferred on the fifth day. In cases of ovarian hyperstimulation syndrome, hydrosalpinx, or endometrial asynchrony, fresh ET was aborted, and all embryos were frozen. Medication for luteal support was provided continuously after ET until pregnancy was either confirmed absent or until approximately 10 weeks of gestation, at which point they were gradually tapered off (16).

The criteria for choosing hCG versus a dual trigger were applied consistently across all COS protocols and were based on individual response and OHSS risk rather than on protocol type.

Although this study spans a 10-year period (2014–2023), the core IVF laboratory procedures at the study center (including fertilization methods, embryo culture conditions, and grading criteria) remained stable throughout. Minor incremental updates in culture media or equipment followed manufacturer recommendations and institutional standard operating procedures and were implemented across all COS protocols simultaneously. Similarly, the fundamental structure of the four COS protocols did not change during the study; any refinements in gonadotropin dosing or trigger choice were individualized clinical decisions and were not systematically protocol-specific. Accordingly, temporal changes are unlikely to have introduced systematic bias favoring any particular protocol.

### Data collection and outcomes

For this study, a range of baseline data was gathered. This information included: The age of the patient and her partner. The length and classification of their infertility. Basal hormone levels, specifically FSH, AMH, estradiol (E2), luteinizing hormone (LH), Body mass index (BMI), and AFC. Any past history of cystectomy. Parameters associated with the COS protocol. The primary outcome was designated as the LBR, with secondary measures including the CPR, the rate of multiple pregnancies, miscarriage, and ectopic pregnancy. For this analysis, the outcomes were defined as follows:

Live birth was defined as the delivery of at least one infant exhibiting signs of life at or after 22 weeks of gestation. Clinical pregnancy was defined as the presence of a gestational sac with fetal cardiac activity on ultrasound. Miscarriage was defined as the loss of a clinically recognized pregnancy before 22 weeks of gestation. Multiple pregnancy was defined as the existence of more than one fetus during a single pregnancy. Cumulative live birth rate (CLBR) was defined as the probability of achieving at least one live birth after all embryo transfers (fresh and frozen) derived from a single COS cycle had been completed.

For each COS protocol, CLBR, live birth rate (LBR), and clinical pregnancy rate (CPR) were calculated using two denominators. First, an intention-to-treat approach was applied, with the denominator defined as all started COS cycles, regardless of whether a fresh embryo transfer took place. Second, outcomes were also calculated per transfer cycle, using only cycles in which a fresh transfer was performed. Per-started-cycle estimates were prespecified as the primary analysis, with per-transfer estimates reported as secondary, descriptive results. All pregnancy outcome rates reported in this study were recalculated using the 22-week threshold for live birth to ensure comparability with international reports.

Because of the retrospective nature of this study and the structure of the electronic medical record system, detailed data on endometriosis stage (e.g., rASRM classification), adenomyosis, endometrioma characteristics (size, laterality, surgical technique), embryonic quality scoring, endometrial thickness at trigger or transfer, specific IVF/ICSI indications, and calendar year of treatment were not systematically recorded in a standardized format and were therefore not available for inclusion in the statistical models.

### Statistical analysis

Statistical analysis was performed using SPSS 26.0 (IBM, Armonk, NY, USA). To determine if continuous variables followed a normal distribution, the Shapiro-Wilk test was used. Data that were normally distributed are shown as mean ± standard deviation (SD) and were compared with an analysis of variance (ANOVA). Data that were not normally distributed are presented as the median with the 25th-75th percentiles and were analyzed with the Kruskal-Wallis test. Categorical variables are reported as frequencies and percentages, and these were analyzed with either the chi-squared test or Fisher’s exact test as needed. Between-group differences in pregnancy outcomes were first evaluated using unadjusted pairwise comparisons with the ultra-long protocol as the reference, and Bonferroni correction was applied for six protocol-to-protocol comparisons (ultra-long vs. long, short, and antagonist; long vs. short; long vs. antagonist; short vs. antagonist). Multivariable logistic regression models were fitted with COS protocol entered as a categorical variable (ultra-long protocol as the reference), adjusting for age, infertility duration, history of cystectomy, basal FSH, LH, E2, AMH, AFC, and BMI to estimate adjusted odds ratios (ORs) for live birth and clinical pregnancy. Covariates for the multivariable logistic regression models were selected based on clinical relevance and univariable associations with the outcomes. Age, infertility duration, history of endometrioma cystectomy, basal FSH, LH, E2, AMH, AFC, BMI, and COS protocol were included *a priori* because of their established or plausible influence on ovarian response and reproductive outcomes. Only cycles with complete data for these variables and pregnancy outcomes were analyzed; there were no missing values for the covariates included in the multivariable models. The variance inflation factor (VIF) was used to evaluate multicollinearity, with a VIF value below 5 indicating an absence of significant multicollinearity. All VIF values were < 5 before or after PSM or IPTW, indicating an absence of problematic multicollinearity and supporting model stability (**Supplementary Table S1**). Throughout all analyses, a two-sided P-value of less than 0.05 was considered to be statistically significant.

Stabilized inverse probability weighting (IPTW)-weighted sensitivity analysis and propensity score matching (PSM) analysis were performed using R (version 4.4.1, R Core Team, 2024), with the necessary computational packages obtained from the CRAN official repository. The statistical significance level was established at α = 0.05.

Because COS protocols were not randomly assigned, confounding by indication related to baseline ovarian reserve and patient characteristics was anticipated. To mitigate this, a multinomial propensity score for the four COS protocols using six covariates was estimated: age, BMI, basal FSH, AMH, AFC, and basal E2. Stabilized inverse probability of treatment weights (IPTW) were then calculated and truncated at the 1st and 99th percentiles to reduce the influence of extreme weights. Covariate balance after weighting was assessed using the maximum standardized mean difference (MaxSMD) across all pairwise protocol comparisons; MaxSMD values < 0.10 were considered well balanced, and values between 0.10 and 0.20 were interpreted as indicating residual imbalance. As a sensitivity analysis, 1:1:1:1 PSM was performed on the same six covariates, generating matched cohorts with balanced ovarian reserve characteristics across protocols. CLBR, LBR, and CPR were calculated per started cycle (intention-to-treat), counting any live birth from fresh or subsequent frozen embryo transfers resulting from that COS cycle. These outcomes were compared across protocols in the crude dataset and then in IPTW-weighted and 1:1:1:1 propensity-score-matched analyses.

## Results

### Patients selection and baseline characteristics

**Figure 1:**
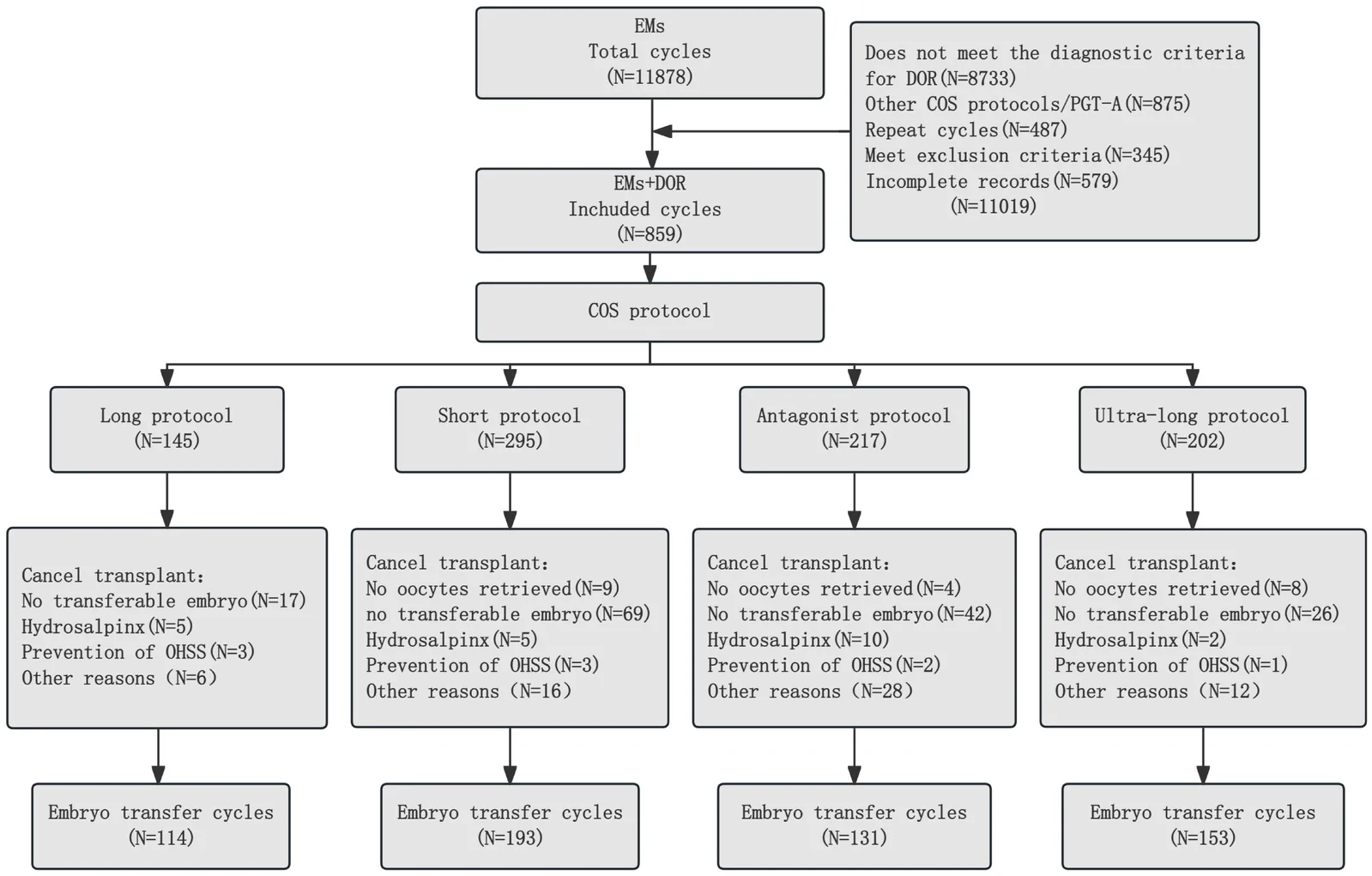
Flowchart of patient selection.

A total of 11,878 ART cycles in women with EM were initially identified. After applying the inclusion and exclusion criteria, 859 women with EM and DOR who initiated COS cycles were included. The initiated cycles, cancellation reasons, and transfer cycles for each protocol are shown in **Figure 1**.

Out of the 859 total cycles in the study, 684 cycles obtained transplantable embryos, and 591 culminated in a fresh embryo transfer (ET). The number of ETs for each protocol group was as follows: long protocol (114 of 145 cycles), short protocol (193 of 295 cycles), antagonist protocol (131 of 217 cycles), and ultra-long protocol (153 of 202 cycles).

As expected in this observational cohort, the four COS protocol groups differed significantly in several baseline ovarian reserve indices, including basal FSH, AMH, AFC, and basal E2 (all P < 0.05, **Table 1**). In the 1:1:1:1 PSM analysis, 101 women were retained in each protocol group, and the MaxSMD across all covariates decreased to 0.052, demonstrating a good balance of key ovarian reserve characteristics. The total amount and duration of Gn administration, hormone levels on the trigger day (E2 and LH), and the number of retrieved oocytes showed significant differences among the four protocols (**Supplementary Table S2**). After applying IPTW based on age, BMI, basal FSH, AMH, AFC, and basal E2, covariate balance improved substantially; however, basal E2 retained a MaxSMD of 0.167, indicating residual imbalance (**Supplementary Table S3**).

**Table 1:** Baseline characteristics and COS-related parameters across the four protocols.

Variable	Long protocol (n=145)	Short protocol (n=295)	Antagonist protocol (n=217)	Ultra-long protocol (n=202)	P
No. of cycles	145	295	217	202	
Age, years	32.00 (29.50–35.00)	32.00 (30.00–36.00)	33.00 (30.00–35.00)	32.00 (30.00–35.00)	0.277
Age of the husband, years	33.00 (30.00–36.00)	33.00 (30.00–36.00)	33.00 (30.00–36.00)	32.50 (30.0–36.00)	0.728
Infertility duration, years	3.00 (2.00–5.00)	3.00 (2.00–5.00)	3.00 (1.50–5.00)	3.00 (2.00–5.00)	0.578
Type of infertility					0.200
Primary infertility	82 (56.6%)	158 (53.7%)	118 (54.4%)	127 (62.9%)	
Secondary infertility	63 (43.4%)	136 (46.3%)	99 (45.6%)	75 (37.1%)	
History of endometrioma cystectomy					0.660
No	83 (57.2%)	151 (51.2%)	115 (53.0%)	104 (51.5%)	
Yes	62 (42.8%)	144 (48.8%)	102 (47.0%)	98 (48.5%)	
Basal FSH, IU/L	7.35 (6.35–8.75) ^a^	8.30 (6.68–10.34)^a^	8.14 (6.40–10.36)	7.82 (6.42–10.52)	0.024
Basal LH, IU/L	4.68 (3.47–5.93)	4.48 (3.41–5.93)	4.44 (3.23–6.22)	4.57 (3.35–6.10)	0.952
Basal E2, pg/mL	37.50 (28.55–50.80)	42.85 (30.70–62.10)	40.13 (28.15–57.70)	40.20 (29.38–63.64)	0.048
AMH, ng/mL	0.94 (0.71–1.11) ^a,b^	0.77 (0.48–1.03) ^a^	0.84 (0.49–1.08)^b^	0.87 (0.47–1.32)	0.001
AFC	7.00 (5.00–11.00) ^a,b,c^	6.00 (4.00–8.00) ^a,e^	5.00 (4.00–7.00) ^b^	5.00 (3.00–7.00) ^c,e^	<0.001
BMI, kg/m^2^	21.99 (20.64–23.99)	22.62 (20.50–24.58)	22.57 (20.89–25.15)	22.73 (20.80–25.25)	0.226
Total amount of Gn, IU	2175.00 (1725.00–2850.00) ^c^	2062.50 (1600.00–3056.25) ^e^	2250.00 (1800.00–3000.00) ^f^	3750.00 (2793.75–4725.00) ^c,e,f^	<0.001
Duration of Gn administration, days	10.00 (9.00–12.00) ^a,b,c^	9.00 (8.00–11.00) ^a,e^	9.00 (8.00–11.00) ^b,f^	12.00 (10.00–14.00) ^c,e,f^	<0.001
E2 on the trigger day, pg/mL	2127.00 (1608.50–2782.50) ^b,c^	1832.50 (1269.25–2603.50) ^d,e^	1266.50 (906.30–1287.25) ^b,d,f^	1559.50 (1013.75–2268.25) ^c,e,f^	<0.001
P on the trigger day, ng/mL	0.75 (0.44–0.98)	0.70 (0.48–0.96) ^d^	0.57 (0.42–0.82) ^d^	0.64 (0.42–0.93)	0.028
LH on the trigger day, IU/L	2.48 (2.00–3.16) ^a,^	5.59 (3.85–7.82) ^a^	3.45 (1.94–6.02)	1.44 (0.94–2.28)	<0.001
No. of follicles ≥14 mm on the trigger day	6.00 (4.00–8.00) ^a,b,c^	4.00 (3.00–6.00) ^a,e^	4.00 (3.00–6.00) ^b,f^	5.00 (3.00–8.00) ^c,e,f^	<0.001
No. of retrieved oocytes	7.00 (4.00–10.00) ^a,b,c^	4.00 (3.00–6.00) ^a^	4.00 (2.00–6.00) ^b^	5.00 (3.00–8.00) ^c^	<0.001
No. of fertilized oocytes	4.00 (2.00–6.00) ^a,b^	3.00 (1.00–4.00) ^a,e^	3.00 (1.00–5.00) ^b^	3.00 (2.00–5.00) ^e^	<0.001
No. of transferred embryos	2.00 (1.00–2.00) ^b^	2.00 (1.00–2.00)	2.00 (1.00–2.00) ^b,f^	2.00 (1.00–2.00) ^f^	0.004
Types of transferred embryos					0.102
Cleavage-stage	98 (86.0%)	148 (76.7%)	110 (84.0%)	130 (85.0%)	
Blastocyst	16 (14.0%)	45 (23.3%)	21 (16.0%)	23 (15.0%)	
No. of frozen embryos	1.00 (0.00–2.00)	1.00 (0.00–1.00)	1.00 (0.00–2.00)	1.00 (0.00–2.00)	0.036
No. of available embryos	3.00 (2.00–4.00)	2.00 (2.00–3.00)	2.00 (2.00–3.00)	2.00 (2.00–3.00)	0.086
No. of no available oocyte cycles	0 (0.0%)	9 (3.1%)	4 (1.8%)	8 (4.0%)	0.061 ^g^
No. of no available embryo cycles	17 (11.7%) ^a^	78 (26.4%) ^a^	46 (21.2%)	34 (16.8%)	0.002

Non-normally distributed variables are presented as median (interquartile range), while categorical variables are presented as n (%).

^a^long vs. short; ^b^long vs. antagonist; ^c^long vs. ultra-long; ^d^short vs. antagonist; ^e^ultra-long vs. short; ^f^ultra-long vs. antagonist; ^g^Fisher’s exact test.

COS, controlled ovarian stimulation; No., number; FSH, follicle-stimulating hormone; LH, luteinizing hormone; E2, estradiol; AMH, anti-Müllerian hormone; AFC, antral follicle count; BMI, body mass index; Gn, gonadotropin; HCG, human chorionic gonadotropin; P, progesterone.

### Outcomes

**Figure 2:**
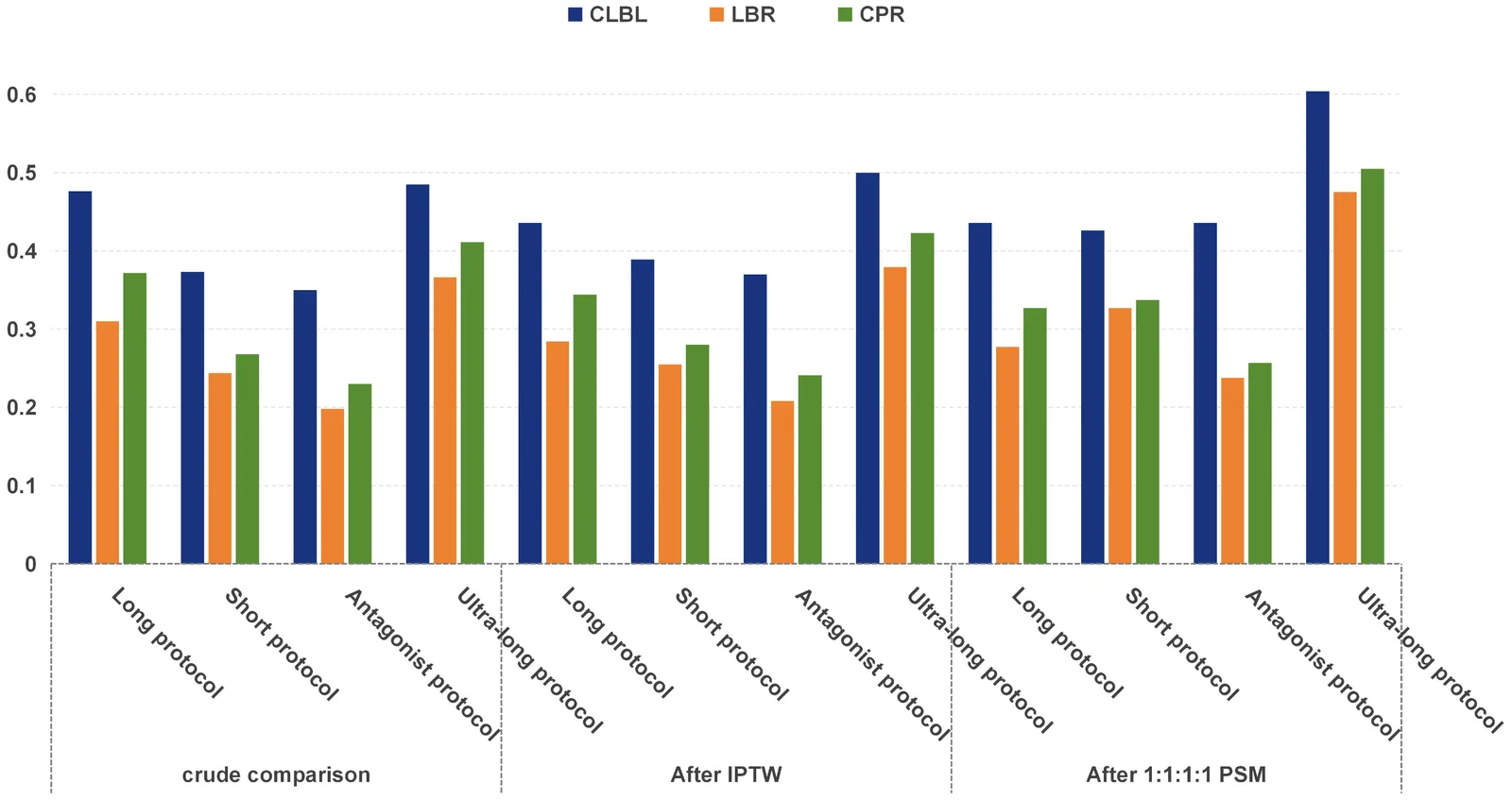
Pregnancy outcomes by controlled ovarian stimulation (COS) protocol. The cumulative live birth rate, live birth, and clinical pregnancy rates were higher in the ultra-long protocol than in the other protocols.

Significant differences in LBRs and CPRs were observed across the four COS protocols per started cycles and fresh ET cycles (all P<0.05) (**Tables 2**, **3**). For started cycles, the ultra-long protocol group demonstrated superior CLBR (48.5%), LBR (36.6%), and CPR (41.1%) compared with the long (CLBR = 47.6%, LBR = 31.0%, CPR = 37.2%), short protocol (CLBR = 37.3%, LBR = 24.4%, CPR = 26.8%), and antagonist protocol (CLBR = 35.0%, LBR = 19.8%, CPR = 23.0%) (**Figure 2**). The pairwise comparisons showed that the ultra-long protocol group had significantly elevated LBRs and CPRs compared with the antagonist protocol group (all P<0.05). For fresh ET cycles, the ultra-long protocol group demonstrated superior LBR (48.4%) and CPR (54.2%) compared with the long (LBR = 39.5%, CPR = 47.4%), short protocol (LBR = 37.3%, CPR = 40.9%), and antagonist protocol (LBR = 32.8%, CPR = 38.2%) (**Figure 2**). The pairwise comparisons showed that the ultra-long protocol group had significantly elevated LBRs and CPRs compared with the antagonist protocol group (all P<0.05).

**Table 2:** Pregnancy outcomes per started cycles across the four protocols.

Variable	Long protocol (n=145)	Short protocol (n=295)	Antagonist protocol (n=217)	Ultra-long protocol (n=202)	P
No. of cycles	145	295	217	202	
LBR	45 (31.0%)	72 (24.4%) ^e^	43 (19.8%) ^f^	74 (36.6%) ^e,f^	0.001
CPR	54 (37.2%)	79 (26.8%) ^e^	50 (23.0%) ^f^	83 (41.1%) ^e,f^	<0.001
Miscarriage rate	14 (9.7%)	12 (4.1%)	10 (4.6%)	12 (5.9%)	0.099
Ectopic pregnancy rate	4 (2.8%)	4 (1.4%)	1 (0.5%)	0 (0.00%)	0.065 ^g^
Multiple pregnancy rate	8 (5.5%)	18 (6.1%)	11 (5.1%)	22 (10.9%)	0.075
CLBR	69 (47.6%)	110 (37.3%) ^e^	76 (35.0%) ^f^	98 (48.5%) ^e,f^	0.007

Categorical variables are presented as n (%).

^g^ Fisher’s exact test.

ET, embryo transfer; LBR, live birth rate; CPR, clinical pregnancy rate; CLBR, cumulative live birth rate.

**Table 3:** Pregnancy outcomes per transfer cycles across the four protocols.

Variable	Long protocol (n=114)	Short protocol (n=193)	Antagonist protocol (n=131)	Ultra-long protocol (n=153)	P
No. of ET cycles	114	193	131	153	
LBR	45 (39.5%)	72 (37.3%) ^e^	43 (32.8%) ^f^	74 (48.4%) ^e,f^	0.049
CPR	54 (47.4%)	79 (40.9%) ^e^	50 (38.2%) ^f^	83 (54.2%) ^e,f^	0.025
Miscarriage rate	14 (12.3%)	12 (6.2%)	10 (7.6%)	12 (7.8%)	0.303
Ectopic pregnancy rate	4 (3.5%)	4 (2.1%)	1 (0.8%)	0 (0.00%)	0.079 ^g^
Multiple pregnancy rate	8 (7.0%)	18 (9.3%)	11 (8.4%)	22 (14.4%)	0.182

Categorical variables are presented as n (%).

^g^ Fisher’s exact test.

ET, embryo transfer; LBR, live birth rate; CPR, clinical pregnancy rate.

After adjusting for covariates, the type of COS protocol was identified as an independent predictor for both CPR (P = 0.017) and LBR (P = 0.025). The odds of achieving a live birth were significantly lower for patients in the long compared with the ultra-long protocol (OR: 0.577, 95% CI: 0.340-0.979, P = 0.042), short (OR: 0.608, 95% CI: 0.389-0.949, P = 0.028), and antagonist (OR: 0.484, 95% CI: 0.295-0.796, P = 0.004) protocol groups. A similar association was observed for CPR, where the short (OR: 0.559, 95% CI: 0.359-0.869, P = 0.010) and antagonist (OR: 0.484, 95% CI: 0.298-0.788, P = 0.004) protocols were linked to lower rates than the ultra-long protocol (**Tables 4**, **5**).

**Table 4:** Multivariable logistic regression analysis of clinical pregnancies.

Variable	B	SE	Wald	P-value	OR	95% CI	95% CI
						Lower	Upper
Age	-0.042	0.024	2.974	0.086	0.959	0.914	1.006
Duration of infertility	-0.007	0.034	0.046	0.831	0.993	0.929	1.061
History of endometrioma cystectomy	-0.361	0.173	4.367	0.037	0.697	0.496	0.978
Basal FSH	0.025	0.031	0.629	0.428	1.025	0.964	1.090
Basal LH	-0.028	0.046	0.373	0.541	0.972	0.888	1.064
Basal E2	-0.001	0.002	0.461	0.497	0.999	0.995	1.002
AMH	0.185	0.114	2.638	0.104	1.203	0.962	1.504
AFC	0.068	0.030	5.267	0.022	1.070	1.010	1.134
COS protocol (vs. ultra-long)			10.185	0.017			
Long protocol	-0.460	0.266	2.985	0.084	0.632	0.375	1.064
Short protocol	-0.582	0.225	6.686	0.010	0.559	0.359	0.869
Antagonist protocol	-0.725	0.248	8.519	0.004	0.484	0.298	0.788
BMI	0.035	0.027	1.676	0.196	1.035	0.982	1.092

B, regression coefficient; SE, standard error; OR, odds ratio; CI, confidence interval; FSH, follicle-stimulating hormone; LH, luteinizing hormone; E2, estradiol; AMH, anti-Müllerian hormone; AFC, antral follicle count; COS, controlled ovarian stimulation; BMI, body mass index.

**Table 5:** Multivariable logistic regression analysis of live births.

Variable	B	SE	Wald	P-value	OR	95% CI	95% CI
						Lower	Upper
Age	-0.064	0.025	6.505	0.011	0.938	0.893	0.985
Duration of infertility	-0.020	0.035	0.324	0.560	0.980	0.916	1.050
History of endometrioma cystectomy	-0.340	0.177	3.714	0.054	0.712	0.504	1.006
Basal FSH	0.024	0.032	0.580	0.446	1.025	0.962	1.091
Basal LH	-0.039	0.048	0.685	0.408	0.961	0.875	1.056
Basal E2	-0.002	0.002	1.205	0.272	0.998	0.994	1.002
AMH	0.208	0.115	3.253	0.071	1.231	0.982	1.543
AFC	0.058	0.030	3.782	0.052	1.060	1.000	1.124
COS protocol (vs. ultra-long)			9.387	0.025			
Long protocol	-0.549	0.270	4.149	0.042	0.577	0.340	0.979
Short protocol	-0.498	0.227	4.807	0.028	0.608	0.389	0.949
Antagonist protocol	-0.725	0.253	8.194	0.004	0.484	0.295	0.796
BMI	0.016	0.027	0.324	0.569	1.016	0.963	1.072

B, regression coefficient; SE, standard error; OR, odds ratio; CI, confidence interval; FSH, follicle-stimulating hormone; LH, luteinizing hormone; E2, estradiol; AMH, anti-Müllerian hormone; AFC, antral follicle count; COS, controlled ovarian stimulation; BMI, body mass index.

After conducting 1:1:1:1 PSM, the CLBR was significantly lower in all three groups (the long, short, and antagonist protocols) when compared to the ultra-long protocol (odds ratio [OR] = 0.49-0.51, p < 0.02). Similarly, the CPR was significantly lower in all three groups compared to the ultra-long protocol (OR = 0.34–0.50, p < 0.02), with the most pronounced difference observed between the antagonist and ultra-long protocol (OR = 0.34, p = 0.0004). Additionally, LBR was significantly lower in all three groups compared to the ultra-long protocol (OR = 0.34-0.54, p < 0.04) (**Supplementary Table S4**).

After applying IPTW, the short and antagonist protocol continued to exhibit significantly lower CLBR compared to the ultra-long protocol (OR = 0.64 and 0.59, both p < 0.05). In terms of CPR, the short protocol demonstrated a significantly lower rate compared to the ultra-long protocol (OR = 0.58, p = 0.017), as did the antagonist protocol (OR = 0.51, p = 0.008). Although the trend for the long protocol compared to the ultra-long protocol was similar, it did not achieve statistical significance (OR = 0.63, p = 0.102). Regarding LBR, both the short and antagonist protocol showed significant differences when compared to the ultra-long protocol (OR = 0.62 and 0.52, both p < 0.05), while the long protocol approached significance (OR = 0.58, p = 0.054) (**Supplementary Table S5**).

In the unadjusted pairwise comparisons with Bonferroni correction, only the antagonist versus ultra-long protocol contrast reached statistical significance for LBR and CPR. After multivariable adjustment for age, ovarian reserve markers, BMI, infertility duration, and history of cystectomy, the long and short protocols also showed significantly lower odds of live birth than the ultra-long protocol. This change reflects both the reduced multiplicity of comparisons in the regression framework and the increased precision obtained by adjusting for key prognostic covariates, rather than the instability of the model.

## Discussion

This research demonstrated that for infertile women with both EM and DOR who were undergoing fresh embryo transfers, the ultra-long protocol yielded the highest LBR at 36.6%, CPR at 41.1%, and CLBR at 48.5%. Both IPTW and PSM demonstrated that the ultra-long protocol was significantly superior to the other protocols across three pregnancy outcomes. In multivariable analyses, the ultra-long protocol was associated with higher odds of clinical pregnancy and live birth than the short and antagonist protocols, and with a modest, borderline-significant advantage over the long protocol. Given that some adjusted estimates (e.g., for the long protocol) have confidence intervals that approach unity and P-values close to 0.05, these differences should be interpreted with appropriate caution. Nevertheless, these findings suggest that the ultra-long protocol may be the most beneficial approach for infertile women with EM and DOR who are pursuing treatment with assisted reproductive technology. The ultra-long protocol may be a promising COS option for women with EM and DOR, but the current results should be interpreted as hypothesis-generating rather than definitive.

The ultra-long protocol is characterized by an extended period of suppression using a GnRH agonist, which is thought to activate GnRH receptors in the pituitary gland. This process suppresses the body’s own GnRH activity, leading to reduced secretion levels of FSH and LH. It prevents premature follicular luteinization, improves oocyte and embryo quality, and may reduce inflammation associated with EM lesions. These effects enhance endometrial receptivity and increase the likelihood of successful embryo implantation (17, 18). Still, the issue in the patients included here (EM and DOR) is that DOR leads to a small yield of oocytes, but the endogenous inhibition of GnRH may lead to a considerable increase in both the time frame and amount of Gn administered when employing the ultra-long protocol. Furthermore, while the ultra-long protocol group presented with a significantly lower AFC and retrieved fewer oocytes than the long protocol group, a direct pairwise comparison between the two showed no significant statistical differences in the numbers of fertilized oocytes, transferred embryos, frozen embryos, or total available embryos. However, after the application of 1:1:1:1 PSM, no significant differences were observed in the aforementioned parameters between the two groups. Nevertheless, the disparity in pregnancy outcomes between the two groups became more pronounced. A study involving 342 women with DOR post-endometrioma cystectomy (19) found that an extended GnRH-agonist regimen may enhance the clinical outcomes of IVF-ET; however, there were no notable differences found when this was compared with other treatment methods.

In the logistic regression analysis, the ultra-long protocol was associated with significantly higher CPRs than the short and antagonist protocols, and it yielded significantly higher LBRs when compared to the long, short, and antagonist protocols. This finding is partially supported by a meta-analysis of international studies by Liu et al. (20), which showed the ultra-long protocol produced a better CPR than the long protocol (RR = 1.31, 95%CI: 1.11-1.55, P = 0.002) in patients with endometriosis, though that study did not specifically address patients with coexisting DOR. In contrast, other research provides conflicting results, with one recent randomized controlled trial indicating that the ultra-long protocol failed to improve the CPR in women with endometriosis when compared to the long protocol. For this reason, any potential benefits of the ultra-long protocol must be carefully weighed against the treatment delays associated with this approach (21). Similar results were reported by Kaponis et al. (22). Of note, those previous studies were performed in patients with EM without a focus on DOR. Furthermore, there are worries about a small oocyte yield when using the ultra-long protocol in patients with EM and DOR (16, 23). After 1:1:1:1 PSM, the COS-related parameters of the ultra-long protocol showed no significant differences compared to the other three protocols, aside from total amount and duration of Gn, and hormone levels on the trigger day. However, CLBR, CPR, and LBR were all significantly superior to those of the other three protocols.

The short protocol demonstrates a transient stimulating effect of GnRH-agonist, accompanied by relatively mild suppression of the pituitary. It maintains LH at elevated levels, increasing early follicle recruitment and ovarian response and enhancing oocyte maturation rate (16). Previous studies from around the globe have shown that the ultra-long protocol is associated with higher CPR and LBR compared to the short protocol in women with EM, even in those with diminished ovarian reserve (16, 18, 23–26). On the other hand, there were no noteworthy differences when comparing the antagonist and short protocols regarding baseline AMH in the present study, overall Gn dosage, length of Gn treatment, and count of follicles measuring ≥14 mm on trigger day. A direct comparison of the antagonist and short protocols showed no significant differences in LH levels on the trigger day, the number of oocytes retrieved or fertilized, the number of ETs, the quantity of available embryos, or in overall pregnancy outcomes. However, the antagonist protocol was associated with lower E2 levels on the trigger day when compared to the other protocols and resulted in fewer ETs than the ultra-long protocol group. It was also noted that while the difference was not statistically significant after the Bonferroni adjustment, the antagonist protocol group trended towards having more frozen embryos than the ultra-long group, a finding potentially linked to a higher cancellation rate of fresh ETs in the antagonist cohort. Earlier studies indicated that using the antagonist protocol alongside frozen embryo transfers could yield similar pregnancy results to those achieved with the ultra-long protocol in individuals with EM and infertility (27). However, obtaining frozen embryos in patients with DOR is challenging. In this study, after combining the pregnancy outcomes of frozen embryo transfers, the CLBR of the antagonist protocol was still significantly lower than that of the ultra-long protocol, both in the crude statistics and after IPTW and PSM.

Nevertheless, this study does have limitations. The limited sample size restricts the applicability of the results and their generalizability. In addition, being a retrospective study, it is impossible to completely eliminate potential selection bias and unconsidered confounding variables, including the surgical techniques used for ovarian cysts. The lack of standardized data on endometriosis stage, adenomyosis, surgical details, embryo quality, endometrial thickness, IVF/ICSI indication, and treatment year is a major limitation. These unmeasured covariates may confound the relationship between COS protocol and reproductive outcomes, so residual confounding is likely despite multivariable adjustment and propensity score methods. The extended study period (2014–2023) introduces the possibility of temporal bias. Over time, incremental changes in IVF laboratory techniques, culture media, equipment, and clinical management may have occurred. At the study center, core laboratory procedures and the overall framework of the four COS protocols remained broadly consistent, and any updates were implemented across protocols. Even so, unmeasured temporal trends could have influenced outcomes independently of the COS protocol and should be considered when interpreting the results. The variables that could be analyzed were limited to those available in the patient charts. While rigorous inclusion and exclusion criteria could minimize bias, conducting large-scale prospective research to substantiate the findings and verify the conclusions is crucial. The CLBR, including fresh and frozen transfers originating from the index COS cycle, could be calculated, which is particularly important for a fair comparison with the antagonist protocol, where a freeze-all strategy is frequently used. Nevertheless, some patients may have received additional treatment or follow-up at other centers, so the CLBR estimates may still be incomplete for a minority of cycles. In addition, the results are derived from a single tertiary center and may not fully capture different COS and embryo transfer strategies used elsewhere. In addition, after IPTW analysis, the MaxSMD for baseline E2 was 0.167, indicating the presence of residual confounding. Furthermore, this analysis only accounted for six core ovarian reserve covariates and did not include factors such as endometrial thickness, male factors, embryo quality scores, previous cycle history, and other variables that may influence pregnancy outcomes. Consequently, the results may still be subject to bias. A key limitation of this study is the potential for confounding by indication. Because COS protocols were selected clinically rather than randomly assigned, baseline ovarian reserve and other clinical features differed significantly across groups, and these differences may influence both protocol choice and reproductive outcomes. Although a multinomial propensity score model incorporating six core ovarian reserve-related covariates (age, BMI, basal FSH, AMH, AFC, and basal E2) was used, and both IPTW and 1:1:1:1 PSM were applied to improve covariate balance, residual confounding remains likely. After IPTW, basal E2 still showed a MaxSMD of 0.167, and the propensity score analyses did not adjust for additional factors such as endometrial thickness, embryo quality, male factor infertility, prior cycle history, and details of endometrioma surgery, all of which may affect pregnancy outcomes. Consequently, the observed superiority of the ultra-long protocol should be interpreted as an association rather than proof of causality, and confirmation in large, prospective or randomized studies is needed. Notably, the ultra-long protocol group presented with the lowest median AFC yet achieved the best pregnancy outcomes, which might suggest protocol selection driven by patient phenotype. However, in the PSM analysis, where AFC and other ovarian reserve indicators were balanced across groups, the ultra-long protocol remained associated with higher CLBR, LBR, and CPR, arguing against this pattern being solely an artifact of baseline AFC differences. Another important consideration is the choice of denominator for outcome rates. Because fresh transfer cancellation rates and reasons differed substantially between protocols (being highest in the antagonist protocol and lowest in the ultra-long protocol), analyses restricted to transferred cycles are subject to informative censoring and may underestimate outcomes in protocols with higher cancellation. To address this, CLBR, LBR, and CPR were reported primarily per started cycle (intention-to-treat), with per-transfer results shown separately. The consistency of the protocol ranking across both denominators supports the robustness of the findings, but the per-started-cycle analyses provide the more conservative and clinically relevant estimates. Another limitation is that embryo-related factors, such as embryo stage at transfer, the number of embryos transferred, and detailed embryo quality scores, were not included in the multivariable or propensity score analyses. These parameters may be influenced by the COS protocol itself and, in turn, affect pregnancy outcomes, so they represent both potential intermediates and confounders. Because standardized embryo quality data were not consistently available across the entire study period, residual confounding by embryo characteristics cannot be excluded and may have biased the estimated differences between protocols. Finally, although adjustment for prognostic covariates reduced residual confounding and improved precision, some protocol comparisons yielded borderline P-values and confidence intervals that nearly included 1.00, underscoring that the findings should be viewed as suggestive rather than definitive.

In conclusion, for women ≤40 years of age with infertility due to EM and DOR, the ultra-long protocol was associated with higher CLBR, LBR, and CPR than the other COS protocols in this retrospective cohort, even when cumulative outcomes, including frozen transfers, were considered. However, protocol selection should remain individualized, and the findings require confirmation in larger, prospective, or randomized studies.

## Data Availability

The original contributions presented in the study are included in the article/**Supplementary Material**. Further inquiries can be directed to the corresponding author.

## Supplementary material

Supplementary Table 1 VIF before and after PSM or IPTW. VIF: variance inflation factor; PSM: propensity score matching; IPTW: stabilized inverse probability weighting; FSH: follicle-stimulating hormone; LH: luteinizing hormone; E2: estradiol; AMH: anti-Müllerian hormone; AFC: antral follicle count; COS: controlled ovarian stimulation; BMI: body mass index.

Supplementary Table 2 COS-related parameters and main pregnancy outcomes per started cycles across the four protocols after 1:1:1:1 PSM. Non-normally distributed variables are presented as median (interquartile range), while categorical variables are presented as n (%). ^a^ long vs. short; ^b^ long vs. antagonist; ^c^ long vs. ultra-long; ^d^ short vs. antagonist; ^e^ ultra-long vs. short; ^f^ ultra-long vs. antagonist. ^g^ Fisher’s exact test. COS: controlled ovarian stimulation; No.: number; FSH: follicle-stimulating hormone; LH: luteinizing hormone; E2: estradiol; AMH: anti-Müllerian hormone; AFC: antral follicle count; BMI: body mass index; Gn: gonadotropin; HCG: human chorionic gonadotropin; P: progesterone; ET: embryo transfer; LBR: live birth rate; CPR: clinical pregnancy rate; CLBR: cumulative live birth rate.

Supplementary Table 3 COS-related parameters and main pregnancy outcomes after IPTW. ^a^ long vs. short; ^b^ long vs. antagonist; ^c^ long vs. ultra-long; ^d^ short vs. antagonist; ^e^ ultra-long vs. short; ^f^ ultra-long vs. antagonist. ^g^ Fisher’s exact test. No.: number; FSH: follicle-stimulating hormone; E2: estradiol; AMH: anti-Müllerian hormone; AFC: antral follicle count; BMI: body mass index; LBR: live birth rate; CPR: clinical pregnancy rate; CLBR: cumulative live birth rate.

Supplementary Table 4 Multivariable logistic regression analysis after 1:1:1:1 PSM. The analysis included age, BMI, basal FSH, AMH, AFC, and basal E2 as covariates. FSH: follicle-stimulating hormone; E2: estradiol; AMH: anti-Müllerian hormone; AFC: antral follicle count; BMI: body mass index; LBR: live birth rate; CPR: clinical pregnancy rate; CLBR: cumulative live birth rate.

Supplementary Table 5 Multivariable logistic regression analysis after 1:1:1:1 IPTW. The analysis included age, BMI, basal FSH, AMH, AFC, and basal E2 as covariates. FSH: follicle-stimulating hormone; E2: estradiol; AMH: anti-Müllerian hormone; AFC: antral follicle count; BMI: body mass index; LBR: live birth rate; CPR: clinical pregnancy rate; CLBR: cumulative live birth rate.
