# Supplementary material for: A retrospective cohort study comparing controlled ovarian stimulation protocols for IVF/ICSI in women with endometriosis and diminished ovarian reserve
Source: Front Endocrinol (Lausanne). 2026 Aug 14;17:1879193. doi: 10.3389/fendo.2026.1879193 (PMC13521843; doi:10.3389/fendo.2026.1879193)
Supplement: Supplementary file 1 [file DataSheet1.pdf]

Supplementary Table S1. VIF before and after PSM or IPTW

	VIF before PSM or IPTW	VIF after PSM	VIF after IPTW
Age	1.094	1.032	1.041
Infertility duration	1.091	--	--
History of endometrioma cystectomy	1.040	--	--
FSH	1.358	1.104	1.111
LH	1.275	--	--
E2	1.050	1.056	1.050
AMH	1.159	1.202	1.144
No. of retrieved oocytes	1.232	--	--
BMI	1.093	1.079	1.066
Protocol	1.095	--	--
AFC	--	1.181	1.104

VIF: variance inflation factor; PSM: propensity score matching; IPTW: stabilized inverse probability weighting; FSH: follicle-stimulating hormone; LH: luteinizing hormone; E2: estradiol; AMH: anti-Müllerian hormone; AFC: antral follicle count; COS: controlled ovarian stimulation; BMI: body mass index.

Supplementary Table S2. COS-related parameters and main pregnancy outcomes per started cycles across the four protocols after 1:1:1:1 PSM.

	Long protocol	Short protocol	Antagonist protocol	Ultra-long protocol	P
No. of cycles	101	101	101	101	
Age, years	32.00 (30.00–35.00)	32.00 (29.00–35.00)	32.00 (30.00–34.00)	32.00 (29.00–35.00)	0.999
Basal FSH, IU/L	7.53 (6.65–8.86)	7.73 (6.52–9.70)	7.89 (6.23–9.43)	7.86 (6.38–10.18)	0.972
Basal E2, pg/mL	40.50 (30.00–58.30)	40.40 (29.84–59.95)	39.00(26.20–55.60)	39.30 (27.50–63.33)	0.559
AMH, ng/mL	0.98 (0.69–1.18)	0.86 (0.61–1.22)	0.91 (0.62–1.31)	0.97 (0.61–1.37)	0.788
AFC	6.00 (4.50–8.00)	6.00 (4.00–8.00)	5.00 (4.00–8.50)	5.00 (4.00–9.00)	0.850
BMI, kg/m^2	21.97 (20.39–23.99)	22.03 (20.20–24.91)	22.17 (20.47–24.55)	22.43 (20.63–24.73)	0.973
Total amount of Gn, IU	2250.00 (1725.00–2993.75) [c]	2025.00 (1518.75–3287.50) [e]	2250.00 (1800.00–2900.00) [f]	3900.00 (2950.00–4725.00) [c,e,f]	<0.001
Duration of Gn administration, days	10.00 (9.50–12.00) [b,c]	10.00 (8.00–11.50) [e]	9.00 (8.00–10.50) [b,f]	13.00 (11.00–14.50) [c,e,f]	<0.001
E2 on the trigger day, pg/mL	1996.00 (1484.50–2681.50) [b]	1983.50 (1272.50–2947.25) [d]	1394.00 (1082.00–2066.00) [b,d]	1784.00 (1015.00–2573.50)	<0.001
P on the trigger day, ng/mL	0.70 (0.44–0.96)	0.74 (0.49–1.02) [d]	0.57 (0.43–0.84) [d]	0.70 (0.46–1.00)	0.197
LH on the trigger day, IU/L	2.61 (2.10–3.38) [a,c]	5.10 (3.38–7.64) [a,d,e]	2.83 (1.86–5.56) [d,f]	1.33 (0.84–2.20) [c,e,f]	<0.001
No. of follicles ≥14 mm on the trigger day	5.00 (4.00–8.00)	4.00 (3.00–7.00)	5.00 (3.00–7.00)	6.00 (3.00–8.00)	0.091
No. of retrieved oocytes	6.00 (4.00–10.00) [a]	4.00 (3.00–7.00) [a]	5.00 (3.00–8.00)	5.00 (3.00–9.00)	0.019
No. of fertilized oocytes	3.00 (2.00–6.00)	3.00 (1.00–5.00)	3.00 (1.00–5.00)	3.00 (2.00–5.00)	0.238
No. of transferred embryos	2.00 (1.00–2.00)	1.00 (0.00–2.00)	1.00 (0.00–2.00)	2.00 (1.00–2.00)	0.161
No. of frozen embryos	0.00 (0.00–2.00)	0.00 (0.00–1.00)	1.00 (0.00–2.00)	0.00 (0.00–2.00)	0.132
No. of available embryos	2.00 (1.00–4.00)	2.00 (1.00–3.00)	2.00 (2.00–3.00)	2.00 (2.00–3.00)	0.569
No. of no available oocyte cycles	0(0.0%)	3(3.0%)	2(2.0%)	2(2.0%)	0.531 [g]
No. of no available embryo cycles	13(12.9%)	24(23.8%)	14(13.9%)	14(13.9%)	0.115
LBR	28 (27.7%) [c]	33 (32.7%) [e]	24 (23.8%) [f]	48 (47.5%) [c,e,f]	0.002

	**Long protocol**	**Short protocol**	**Antagonist protocol**	**Ultra-long protocol**	**P**
CPR	33 (32.7%) [c]	34 (33.7%) [e]	26 (25.7%) [f]	51 (50.5%) [c,e,f]	0.002
CLBR	44 (43.6%) [c]	43 (42.6%) [e]	44 (43.6%) [f]	61 (60.4%) [c,e,f]	0.030

Non-normally distributed variables are presented as median (interquartile range), while categorical variables are presented as n (%).

[a] long vs. short; [b] long vs. antagonist; [c] long vs. ultra-long; [d] short vs. antagonist; [e] ultra-long vs. short; [f] ultra-long vs. antagonist.

[g] Fisher's exact test

COS: controlled ovarian stimulation; No.: number; FSH: follicle-stimulating hormone; LH: luteinizing hormone; E2: estradiol; AMH: anti-Müllerian hormone; AFC: antral follicle count; BMI: body mass index; Gn: gonadotropin; HCG: human chorionic gonadotropin; P: progesterone; ET: embryo transfer; LBR: live birth rate; CPR: clinical pregnancy rate; CLBR: cumulative live birth rate.

Supplementary Table S3. COS-related parameters and main pregnancy outcomes after IPTW

	Long protocol	Short protocol	Antagonist protocol	Ultra-long protocol	P	MaxSMD
No. of cycles	136.6	293.3	217.0	203.6		
Age, years	8.27 (6.41–10.71)	8.23 (6.35–10.65)	8.26 (6.39–10.72)	8.25 (6.38–10.69)	0.974	0.041
Basal FSH, IU/L	0.82 (0.43–1.42)	0.77 (0.38–1.37)	0.75 (0.36–1.35)	0.74 (0.35–1.33)	0.900	0.079
Basal E2, pg/mL	6.00 (4.00–8.00)	6.00 (4.00–8.00)	6.00 (4.00–8.00)	6.00 (3.00–9.00)	0.880	0.086
AMH, ng/mL	41.20 (28.00–59.80)	43.00 (29.00–64.50)	42.80 (28.20–63.00)	43.50 (29.50–65.50)	0.167	0.165
AFC	32.09 (29.76–34.42)	32.49 (29.80–35.18)	32.55 (30.20–35.00)	32.51 (30.05–34.97)	0.712	0.131
BMI, kg/m^2	22.94 (20.68–25.20)	23.02 (20.74–25.30)	23.05 (20.88–25.22)	23.05 (20.84–25.26)	0.993	0.036
LBR	38.8 (28.4%)	74.7 (25.5%) [e]	45.2 (20.8%) [f]	77.1 (37.9%) [e,f]	0.002	-
CPR	47.0 (34.4%)	82.2 (28.0%) [e]	52.3 (24.1%) [f]	86.1 (42.3%) [e,f]	0.002	-
CLBR	59.5 (43.6%)	114.0 (38.9%) [e]	80.3 (37.0%) [f]	101.9 (50.0%) [e,f]	0.049	-

[a] long vs. short; [b] long vs. antagonist; [c] long vs. ultra-long; [d] short vs. antagonist; [e] ultra-long vs. short; [f] ultra-long vs. antagonist.

[g] Fisher's exact test

No.: number; FSH: follicle-stimulating hormone; E2: estradiol; AMH: anti-Müllerian hormone; AFC: antral follicle count; BMI: body mass index; LBR: live birth rate; CPR: clinical pregnancy rate; CLBR: cumulative live birth rate.

Supplementary Table S4. Multivariable logistic regression analysis after 1:1:1:1 PSM

	**B**	**SE**	**Wald**	**P-value**	**OR**	**95% CI**	
						**Lower**	**Upper**
CLBR							
COS protocol (vs. ultra-long)							
Long protocol	-0.681	0.286	-2.383	0.017	0.506	0.289	0.886
Short protocol	-0.721	0.286	-2.52	0.012	0.486	0.277	0.852
Antagonist protocol	-0.681	0.286	-2.383	0.017	0.506	0.289	0.886
CBR							
COS protocol (vs. ultra-long)							
Long protocol	-0.743	0.291	-2.554	0.011	0.476	0.269	0.841
Short protocol	-0.698	0.290	-2.41	0.016	0.498	0.282	0.878
Antagonist protocol	-1.079	0.302	-3.57	<0.001	0.340	0.188	0.615
LBR							
COS protocol (vs. ultra-long)							
Long protocol	-0.859	0.299	-2.878	0.004	0.424	0.236	0.760
Short protocol	-0.624	0.291	-2.144	0.032	0.536	0.303	0.948
Antagonist protocol	-1.067	0.307	-3.473	<0.001	0.344	0.188	0.628

The analysis included age, BMI, basal FSH, AMH, AFC, and basal E2 as covariates. FSH: follicle-stimulating hormone; E2: estradiol; AMH: anti-Müllerian hormone; AFC: antral follicle count; BMI: body mass index; LBR: live birth rate; CPR: clinical pregnancy rate; CLBR: cumulative live birth rate.

Supplementary Table S5. Multivariable logistic regression analysis after 1:1:1:1 IPTW

	B	SE	Wald	P-value	OR	95% CI Lower	Upper
**CLBR**							
COS protocol (vs. ultra-long)							
Long protocol	-0.260	0.244	-1.065	0.287	0.771	0.477	1.245
Short protocol	-0.454	0.195	-2.328	0.020	0.635	0.433	0.931
Antagonist protocol	-0.533	0.209	-2.551	0.011	0.587	0.389	0.884
**CBR**							
COS protocol (vs. ultra-long)							
Long protocol	-0.455	0.278	-1.636	0.102	0.635	0.368	1.096
Short protocol	-0.548	0.229	-2.388	0.017	0.578	0.368	0.907
Antagonist protocol	-0.666	0.252	-2.643	0.008	0.514	0.313	0.843
**LBR**							
COS protocol (vs. ultra-long)							
Long protocol	-0.544	0.282	-1.931	0.054	0.58	0.334	1.009
Short protocol	-0.475	0.231	-2.055	0.04	0.622	0.395	0.979
Antagonist protocol	-0.663	0.256	-2.587	0.01	0.515	0.311	0.852

The analysis included age, BMI, basal FSH, AMH, AFC, and basal E2 as covariates. FSH: follicle-stimulating hormone; E2: estradiol; AMH: anti-Müllerian hormone; AFC: antral follicle count; BMI: body mass index; LBR: live birth rate; CPR: clinical pregnancy rate; CLBR: cumulative live birth rate.
